# Photocleavable linker for the patterning of bioactive molecules

**DOI:** 10.1038/srep18309

**Published:** 2015-12-16

**Authors:** Seraphine V. Wegner, Oya I. Sentürk, Joachim P. Spatz

**Affiliations:** 1 Department of New Materials and Biosystems, Institution Max Planck Institute for Intelligent Systems, Heisenbergstr. 3, 70569 Stuttgart, Germany; 2 Department of Biophysical Chemistry, University of Heidelberg, Im Neuenheimer Feld 253, 69120 Heidelberg, Germany

## Abstract

Herein, we report the use of a versatile photocleavable nitrobenzyl linker to micropattern a wide variety of bioactive molecules and photorelease them on demand. On one end, the linker has an NHS group that can be coupled with any amine, such as peptides, proteins or amine-linkers, and on the other end an alkyne for convenient attachment to materials with an azide functional group. This linker was conjugated with NTA-amine or the cell adhesion peptide cRGD to enable straightforward patterning of His6-tagged proteins or cells, respectively, on PEGylated glass surfaces. This approach provides a practical way to control the presentation of a wide variety of bioactive molecules with high spatial and temporal resolution. The extent of photocleavage can also be controlled to tune the biomolecule density and degree of cell attachment to the surface.

The controlled presentation, patterning and release of bioactive molecules such as adhesion peptides and proteins on surfaces is important for the development of biomaterials that can guide cell behavior^1^. A key motivation to micropattern such bioactive molecules on surfaces and controllably detach them *in vitro* is to mimic the patterns and dynamics created during complex multicellular tissue formation *in vivo*. To turn on and off interactions dynamically, materials that react to stimuli, such as changes in temperature^2^, electric fields^3^,^4^,^5^, light^6^,^7^,^8^ or biochemical signals^9^,^10^,^11^, have been established^1^,^12^. Photoresponsive materials have been of particular interest as light provides external regulation with the highest accuracy in space and time, is non-invasive, can be used to pattern unconventional geometries (including both 2D and 3D), tune interactions and form gradients by only partial switching^6^,^13^,^14^.

The nitrobenzyl group, which undergoes selective bond cleavage upon irradiation with UV-light, has been a valuable building block to control material properties and the presentation of biomolecules^6^,^13^,^14^. Despite limitations of the nitrobenzyl group, which include slow reaction kinetics, low photochemical efficiency and irradiation with UV-light that can be damaging to cells, it has been used extensively to control cell material interactions^6^,^13^,^14^. To this end, the nitrobenzyl group has been integrated into linkers to attach protein-repellent PEG (poly ethylene glycol) coatings to different types of surfaces and to later locally release the coatings to obtain patches for cell adhesion^15^,^16^. The nitrophenethyl and nitrobenzyl photoremovable groups have also been used as a caging group on the amide back bone or the aspartic acid side chain of the cell adhesion peptide RGD (arginine glycine aspartic acid) to block integrin-mediated cell adhesion and to turn on cell adhesion upon illumination^17^,^18^,^19^. Conversely, the RGD motif has also been attached to PEG coatings through a nitrobenzyl linker to liberate the peptide upon illumination and render parts of the surface non-adhesive^20^. Further, nitrobenzyl groups have been introduced as crosslinkers into hydrogels to control their properties, degradation, the release of proteins from them and cell behaviour with light^21^,^22^,^23^. Such platforms have enabled the patterning of cells in confined and complex geometries and the study of the effect of geometric restrains on cell migration on glass substrates without introducing mechanical damage^24^,^25^. Additionally, the high temporal control provided by such light activated cell adhesion platforms has been exploited to study the role of cell adhesion in myogenic cell differentiation in culture^26^ and inflammation and vascularization *in vivo*^27^.

The patterning of proteins while preserving their activity is equally important for bioanalytical and biomedical applications. The binding of His6-tagged proteins to Ni^2+^-NTA (nitrilotriacetic acid) functionalized materials is a widely used approach for immobilization. Nitrobenzyl groups have been used to tune the interaction between His-tagged proteins and materials functionalized with Ni^2+^-NTA (nitrilotriacetic acid) and pattern His6-tagged proteins and protein complexes^28^,^29^,^30^,^31^. To achieve this the nitrobenzyl group has been incorporated into a photocleavable oligohistidine peptide backbone that can block the Ni^2+^-NTA complex on the surface and can be locally photocleaved for His6-tagged proteins to bind. While these examples provide an elegant ways to pattern His6-tagged proteins the synthesis of these peptides is challenging and it is not possible to release proteins from the surface. To overcome these limitations a complementary set-up where the Ni^2+^-NTA group can be cleaved from the surface selectively is desirable.

While all the above listed examples provide elegant platforms to micropattern adhesion peptides, cells and proteins, they are quite specific to a certain application. To facilitate the micropatterning of different bioactive molecules on materials and use them for cell studies, it is of interest to develop a generalized linker that can be easily conjugated to a wide variety of materials using click-chemistry and can be used to pattern a wide variety of bioactive molecules.

## Results and Discussion

**Figure 1:**
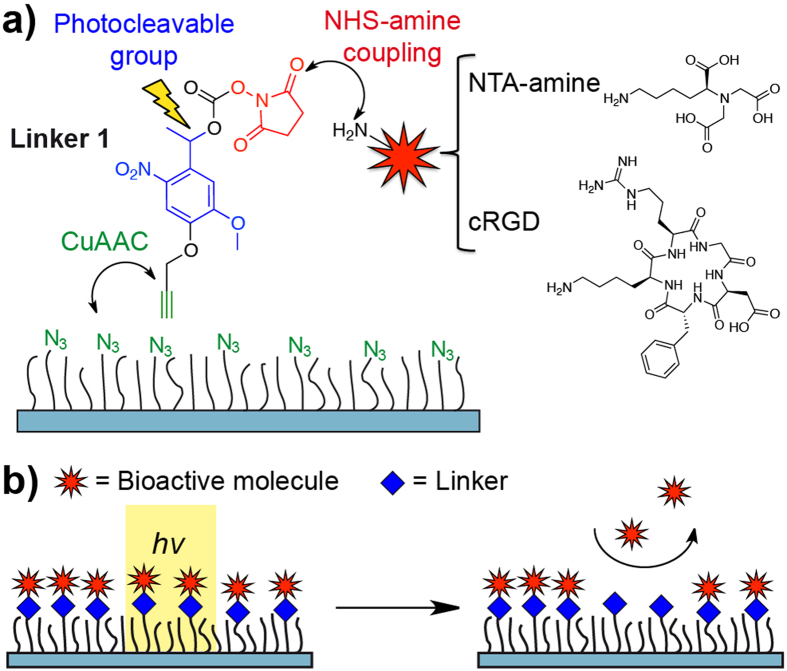
General functionalization strategy. (**a**) Bioactive molecules with an amine such as cRGD and NTA-amine can be linked through the NHS moiety and the molecule can be attached to any material with an azide functional group through CuAAC. The nitrobenzene in the linker allows for the photochemical detachment. (**b**) Surfaces functionalized with bioactive molecules through linker 1 can be pattered by locally cleaving off the molecules with light.

In this article, we present a versatile and convenient strategy to decorate materials with biologically active molecules through a nitrobenzyl linker that can be cleaved on demand. Considering the wide range of amine functional groups in bioactive molecules and the numerous materials with azide functional groups as points of attachment, we synthesized the nitrobenzyl linker 1 as reported in the literature^16^. As shown in Fig. 1a, the linker 1 can be photocleaved with UV light (365 nm), possesses an NHS-ester (N-Hydroxysuccinimide ester) functional group to react with an amine of choice, and possesses an alkyne group for convenient attachment to any material with an azide functional group by using CuAAC (copper catalysed azide alkyne cycloaddition, also known as the “click reaction”). In previous reports linker 1 has been used to connect azide PEG molecules to amine-functionalized surfaces and release the PEG on demand to obtain the cell adhesive starting amine surface^16^. Here, however we envision to use linker 1 to attach a variety of bioactive molecules to passivating PEG coatings and liberate these to obtain inert surfaces. Most bioactive molecules and numerous linkers used for further functionalization methods possess amines, notably peptides, proteins and antibodies. To showcase the wide range of possible applications we coupled linker 1 to NTA-amine, a group that specifically interacts with His-tagged proteins or to the cell adhesion peptide cRGD (cyclic *L*-arginine glycine *L*-aspartic acid *D*-phenylalanine *L*-lysine), to yield NTA-NO_2_ and cRGD-NO_2_, respectively. Later, these molecules are immobilized on glass surfaces coated with a PEG-azide (a PEG3000 with an azide and a triethoxy-silane terminal group, Supplementary Fig. S1)^32^. The PEG coating is protein and cell repellent and the functionalization density with the conjugates of linker 1 can be tuned by adding non-reactive PEG2000 (a PEG2000 with a methoxy and a triethoxy-silane terminal group) during the coating reaction^33^. The patterning of the bioactive molecules is then realized by locally irradiating the surface in the geometry of interest and locally releasing the bioactive active molecule from the surface while the rest of the linker stays on the surface (Fig. 1b).

**Figure 2:**
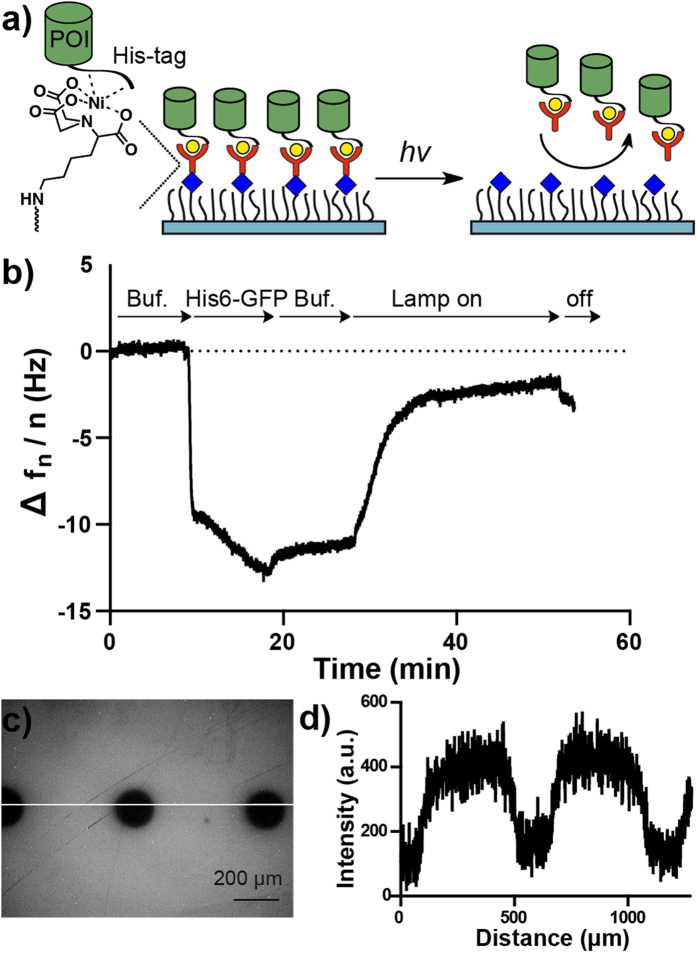
Characterization of NTA-NO_2_. (**a**) Working model of NTA-NO_2_. The His6-tagged POI (protein of interest) binds to the Ni^2+^-NTA-NO_2_ complex presented at the surface. Upon illumination the NTA group is cleaved from the surface and the protein is released. (**b**) His6-GFP binds on SiO_2_ QCM crystals coated with 10 mol% PEG-azide and modified with NTA-NO_2_. When the lamp is turned on the His6-GFP is liberated from the surface. (**c**) Fluorescence image of NTA-NO_2_ modified surface with circular micropatterns (160 μm) and (**d**) the line profile along the line. The surface was illuminated under an upright fluorescence microscope with an adjustable field aperture. The surface was incubated afterwards with Ni^2+^ and His6-GFP for visualization.

As a first demonstration of our platform, we used the NTA-conjugate of linker 1, NTA-NO_2_, to pattern His6-tagged proteins (Fig. 2a). To characterize the photocleavage of the NTA-NO_2_ linker, we functionalized SiO_2_ QCM (quartz crystal microbalance) crystals with 10 or 100 mol% PEG-azide coatings, conjugated NTA-NO_2_ using the CuAAC to these and then loaded the NTA groups with NiCl_2_. Subsequently, the binding of His6-GFP to these surfaces and its release upon UV-irradiation was monitored in real time with QCM. It should be also noted that the UV-illumination causes an increase of a few Hz in the frequency, which is completely reversed when the illumination is stopped. In the case of crystals coated with 10 mol% PEG-azide, 75% of the previously bound His6-GFP is cleaved off after irradiation for 20 minutes and 60% of the His6-GFP is cleaved off from the surfaces with 100 mol% PEG-azide coating under the same conditions (Fig. 2b, Supplementary Fig. S2). It is possible to control the amount of protein release by controlling the exposure time to UV-light. From the QCM measurement it can be seen that about half of the bound His6-GFP is released form 10 mol% PEG-azide surfaces after 3 minutes of irradiation. The incomplete release of the protein could potentially be due to the low photochemical efficiency and slow kinetics observed for nitrobenzyl groups^13^,^14^. His6-GFP was also patterned on glass surfaces with the same set up. For this purpose a 100 mol% PEG-azide coated surface was modified as described above and illuminated under a microscope with a circular pattern (Fig. 2c). Later the surface was first incubated with NiCl_2_ and then His6-GFP. As can be seen from the intensity profile, His6-GFP does not bind to the illuminated areas but to the surrounding space (Fig. 2d). The only reported platform to photopattern His-tagged proteins is based on photocleavable oligohistidine peptides^28^,^29^,^30^,^31^. This approach selectively turns on protein binding to Ni^2+^-NTA sites upon illumination so that His-tagged proteins can be patterned in an oriented manner and allows for the patterning of multiple proteins, but the synthesis of such photocleavable peptides is rather complicated. Our approach is complementary to this existing platform, since we cleave the His-tag-binding Ni^2+^-NTA group from the material to achieve protein patterns. The linker can be photocleaved either before or after the binding of His6-tagged proteins to generate protein patterns. Therefore, this platform is not only useful to form protein micropatterns but also to photo-trigger protein release from these. This can potentially be used to study cell interactions with protein micropatterns and the uptake of immobilized proteins upon photo-cleavage.

**Figure 3:**
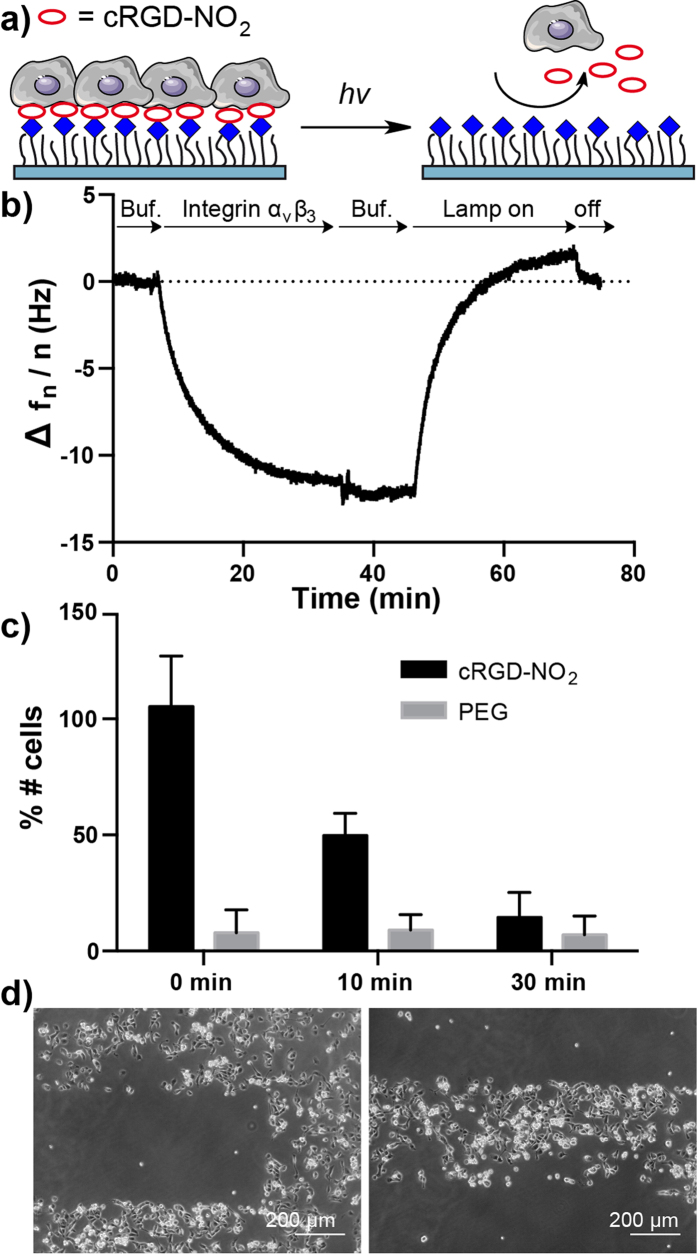
Characterization of cRGD-NO_2_. (**a**) Working model of cRGD-NO_2_. Cells can adhere to cRGD-NO_2_ modified PEG surfaces through integrin binding. Upon illumination the integrin ligand cRGD is cleaved off and cells can no longer adhere on the PEG coating. (**b**) QCM measurements showing integrin α_v_β_3_ binding to cRGD-NO_2_ modified 1 mol% PEG-azide surfaces. The bound integrin α_v_β_3_ is washed off as the surface is irradiated (λ = 365 nm). (**c**) REF cell adhesion on surfaces with 1% PEG-azide coating modified with cRGD-NO_2_. Surfaces are irradiated with light of λ = 365 nm for 0, 10 and 30 minutes to achieve various extends of cRGD cleavage. Unmodified surfaces with the same PEG coating are used as controls and the number of cells on the surface are given as the percentage of cells that adheres on a cRGD modified surface. The error bars are the standard deviation from three independent experiments. (**d**) Patterned MDCK cells on cRGD-NO_2_ modified surfaces. The surface was illuminated under an upright fluorescence microscope with an adjustable field aperture.

As a second case study, we conjugated linker 1 to the adhesion peptide cRGD to yield cRGD-NO_2_ (Fig. 3a). cRGD is recognized by the integrin α_v_β_3_ on the cell surface and mediates cell adhesion. In QCM measurements integrin α_v_β_3_ clearly binds to surfaces with 1 mol% PEG-azide coupled to cRGD-NO_2_ and the bound integrin dissociates completely from the surface upon irradiation with light (λ = 365 nm) for 20 minutes (Fig. 3b). In comparison, the binding of integrin α_v_β_3_ is not reversed with irradiation when the peptide is linked through a non-photocleavable linker (Supplementary Fig. S3). This result excludes the possibility that the UV-light damages the coating and causes dissociation. In previous studies we showed that 1 mol% functionalization with cRGD is sufficient to sustain the adhesion and spreading of REF cells (rat embryonic fibroblast) on this type of PEG coatings^32^. Thus, we also used 1 mol% PEG-azide in all cell experiments to study cell adhesion behavior on cRGD-NO_2_ surfaces, which had been illuminated for various durations with a 365 nm light source (Fig. 3c). Before illumination as many REF cells adhere on the cRGD-NO_2_ surface as on the positive control surface with cRGD. Depending on the initial illumination time, the number of REF cells that can adhere to the cRGD-NO_2_ surface afterwards decreases gradually; if cells are seeded after 10 minutes of illumination only about half as many cells adhered to the surface as before illumination and after 30 minutes the number of cells that attached is comparable to the PEG-coating control. It also should be noted that the illumination of PEG-coated control surfaces did not impair the cell-repellent properties of the coating. Using cRGD-NO_2_ functionalized surfaces it is also possible to pattern adhesive and non-adhesive domains on the surface. Here, nonadhesive stripes are patterned under the microscope and MDCK (Madin-Darby canine kidney) epithelial cells are seeded on the surface (Fig. 3d). The MDCK cells only adhere on the stripes that were not illuminated and on the zones around the pattern but do not adhere on the illuminated regions.

In summary, we demonstrated that linker 1 is a convenient tool to attach different bioactive molecules such as peptides and His6-tagged proteins to materials with azide functional groups as points of attachment and that these biomolecules can be cleaved on demand in a desired pattern non-invasively. While in this study we couple NTA-amine and cRGD to linker 1, this can be extended to many other biomolecules of interest that have amine functional groups such as antibodies, biotin-amine and proteins. The amount of bioactive molecules that are released from the surface can be tuned by controlling the illumination time (or light intensity) as is shown in QCM measurements and the cell adhesion experiments and this can potentially be used to form gradients of these biomolecules.

## Methods

### Synthesis of NTA-NO_2_

Linker 1 (1 eq., 19.6 mg, 50 μmol)^16^ dissolved in 850 μL DMF is added to *N*_α_*,N*_α_-bis(carboxymethyl)-L-lysine hydrate (1 eq., 13.2 mg, 50 μmol) dissolved in 850 μL 0.4 M of Na_2_CO_3_ pH 8.5 buffer. This mixture is kept at room temperature for 24 h and then lyophilized. The remaining solid is dissolved in water and extracted with ethyl acetate. The ethyl acetate phase is evaporated under vacuum and the remaining solid is purified by reverse phase HPLC (A: H_2_O, 0.1% TFA, B: acetonitrile, 0.1% TFA, gradient 20% to 80% B). HR-MS(ESI^–^): [M-2H+K]^–^ observed: *m/z* = 576.11367, calc.: *m/z* = 576.12373. The concentration of the final product is determined to be 7 mM by UV-Vis (ε_350_ = 5000 M^−1^ cm^−1^)

### Synthesis of cRGD-NO_2_

To a solution of linker 1 (2 eq., 2.6 mg, 6.6 μmol)^16^ in 120 μL DMF, a solution of cRGD (1 eq., 2.0 mg, 3.3 μmol) in 100 μL H_2_O and 60 μl of 1 M of Na_2_CO_3_ (pH 8.5) is added and the reaction mixture is kept at room temperature for 24 h. The reaction mixture is lyophilized and the remaining solid is purified by reverse phase HPLC (A: H_2_O, 0.1% TFA, B: Acetonitrile, 0.1% TFA, gradient 20% to 60% B). HR-MS(ESI^+^): [M+Na]^+^ observed: *m/z* = 903.36388, calc.: *m/z* = 903.36075. The concentration of the final product is determined to be 1.2 mM by UV-Vis (ε_350_ = 5000 M^−1^ cm^−1^)

### Synthesis of PEG-azide

PEG-azide is synthesized following a similar protocol as described in previous publications for PEG2000^32^,^33^. To a suspension of amino-terminated PEG-azide (azide-PEG3000-NH_2_, 1 eq., 500 mg, 1.67 mmol, Iris Biotech GmbH) in 1 ml DMF 3-(triethoxysilyl)-propylisocyanate (1.1 eq., 47.74 mg, 1.83 mmol) is added and the reaction is stirred under an argon atmosphere for 24 h at room temperature. The reaction solution is cooled to 0 °C, an excess of diethyl ether (10 ml) is added and the suspension is stirred for 1 h at 0 °C. The precipitate is filtered off, the product is washed with cold diethyl ether and the product is dried under vacuum. Yield: 540 mg, 1.66 mmol, ≈100% ^1^H NMR (300 MHz, CDCl_3_, 25 °C, TMS): δ = 3.89−3.28 (m, ≈350 H, OCH_2_ and NCH_2_), 3.17 (br s, 2 H, SiCH_2_CH_2_CH_2_), 1.68 (br s, 2 H, SiCH_2_CH_2_CH_2_), 1.25 (t, 9 H, CH_3_), 0.65 (br s, 2 H, SiCH_2_).

### PEGylation of SiO_2_ surfaces

Glass slides (20 × 20 mm) are cleaned in freshly prepared Piranha solution (conc. 3:1 H_2_SO_4_:H_2_O_2_ (30%)) for 1 h, rinsed 3 times with Milli-Q water and dried in an N_2_ stream. The SiO_2_ coated QCM crystals are cleaned with a 2% SDS solution overnight, rinsed 3 times with Milli-Q water and dried in an N_2_ stream. The QCM crystals are then are treated with oxygen plasma (TePla 100-E, 0.4 mbar, 150 W, 10 min). For the PEGylation reaction, surfaces are immersed in a solution of appropriate PEG-azide and PEG2000 ratios (13.1 mg PEG2000 and 0.2 mg PEG-azide for 1% PEG-azide, 10.8 mg PEG2000 and 1.8 mg PEG-azide for 10% PEG-azide) and a drop of dry triethylamine in dry toluene (dried over molecular sieves (3 Å)) and kept at 80 °C overnight under a N_2_ atmosphere. The surfaces are first washed with ethyl acetate for 5 minutes by sonication, then with methanol for 5 minutes by sonication and dried in a N_2_ stream.

### CuAAC Reaction on surfaces

The PEG-coated surfaces are incubated in contact with 50–100 μl of reaction solution containing 100 mM L-ascorbic acid, 100 mM Tris HCl (pH 8.5), 150 μM of the alkyne and 1 mM CuSO_4_ in a moisture chamber for 2 hours. Afterwards the surfaces are washed with water and dried under N_2_ stream. Alkynes used in this study include NTA-NO_2_ for QCM measurements and fluorescence measurements, and cRGD-NO_2_ and cRGD alkyne for QCM measurements and cell adhesion studies. The surfaces functionalized with NTA-NO_2_ are washed with: 1) 50 mM EDTA (pH 7.4) for 5 min 2) Buffer A (50 mM Tris HCl (pH 7.4), 300 mM NaCl) for 5 min (2 times). 3) 0.1 M NiCl_2_ for 5 min. 4) Buffer A for 5 min. (2 times).

### QCM (quartz-crystal microbalance) measurements

All QCM measurements are performed on a Q-Sense E4 system (Q-Sense) with SiO_2_ crystals (Q-sense) in a window module (Q-sense), which enabled *in situ* radiation with a 354 nm light source. All measurements are performed with a flow rate of 100 μl/min and at room temperature. QCM experiments performed with NTA-NO_2_ functionalized crystals consist of the following steps: 1) Buffer A as baseline (50 mM Tris HCl (pH 7.4), 300 mM NaCl), 2) 10 minutes 10 μM His6-GFP in Buffer A, 3) 10 minutes Buffer A, 4) 20 minutes UV-light with Buffer A, 5) 10 minutes Buffer A after UV exposure.

QCM experiments for investigating integrin α_v_β_3_ binding to surfaces with cRGD-NO_2_ or cRGD and their photo-cleavage consist of the following steps: 1) Buffer B as baseline (50 mM Tris HCl (pH 7.4), 150 mM NaCl, 2 mM mgCl_2_, 1 mM MnCl_2_), 2) 30 minute incubation with 10 μg/ml integrin α_v_β_3_ in Buffer B, 3) 10 minute Buffer B, 4) 20 minutes UV-light with Buffer B, 5) 10 minutes Buffer B after UV exposure.

### Photopatterning

PEGylated surfaces clicked with NTA-NO_2_ (10:100 PEG-azide and PEG2000) or cRGD-NO_2_ (1:100 PEG-azide and PEG2000), are patterned using the Leica DM6000B microscope and a 20× air lens with the adjustable field aperture. Surfaces are exposed to light using the DAPI filter for 0.5–30 sec and then used for subsequent experiments. NTA-NO_2_ functionalized surfaces are: 1) Washed with buffer A, 2) incubated with 0.1 M NiCl_2_ for 5 minutes, 3) washed with buffer A for 5 minutes, and 4) incubated with 10 μM His6-GFP for 20 minutes. Images are acquired with a Leica DM6000B under a 10× (HC PL APO 10×/0.40 Leica, Wetzlar, Germany) air lens. MDCK cells are seeded on cRGD-NO_2_ functionalized surfaces as described below.

### Cell culture experiments

REF (rat embryonic fibroblasts) (kindly provided by B. Geiger, The Weizmann Institute of Science, Israel) and MDCK (Madin Darby Canine Kidney) epithelial cells are cultured in DMEM supplemented with 10% fetal bovine serum, 1% L-glutamine and 1% penicillin- streptomycin at 37 °C and 5% CO_2_. Surfaces used in cell experiments are washed with sterile PBS at room temperature and cells are cultured in serum-free medium during experiments on functionalized surfaces. Prior to seeding on the surfaces, cells are washed two times with 5 mL of sterile PBS, treated for 5 min with 2 mL of accutase at 37 °C and 5% CO_2_ and then resuspended in in serum-free medium. REF cells are seeded onto samples at a density of ca. 5000 cell/cm^2^ and incubated for 2 h at 37 °C and 5% CO_2_ before fixing the cells with 4% paraformaldehyde (PFA) in PBS (pH 7.4) for 20 min. Samples are washed twice with PBS and mounted in Mowiol containing DAPI (1 μg/mL). Fluorescence images are acquired on a Leica DM6000B microscope (Leica Microsystems, Wetzlar, Germany) equipped with a Leica DFC 365 FX camera. A 10 × 10 tile scan is taken of each surface with a 10× (HC PL APO 10×/0.40 Leica, Wetzlar, Germany) air lens. The number of nuclei in the DAPI channel is analyzed with ImageJ 1.45s (http://imagej.nih.gov/ij) to calculate the number of cells per mm^2^ in comparison to a cRGD-coated surface. The error is given as the standard deviation of three independent experiments. MDCK cells are seeded at 3.5 × 10^5^ cell/cm^2^ on to photopatterned surfaces and incubated for 2 hours at 37 °C and 5% CO_2_. The surfaces are washed twice with PBS to remove unattached cells and are then imaged.

## Additional Information

**How to cite this article**: Wegner, S. V. *et al.* Photocleavable linker for the patterning of bioactive molecules. *Sci. Rep.* **5**, 18309; doi: 10.1038/srep18309 (2015).

## Supplementary Material

Supplementary Information
