# Supplementary material for: Photocleavable linker for the patterning of bioactive molecules
Source: Sci Rep. 2015 Dec 16;5:18309. doi: 10.1038/srep18309 (PMC4680943; doi:10.1038/srep18309)
Supplement: Supplementary Information [file srep18309-s1.pdf]

Photocleavable linker for the patterning of bioactive molecules

Seraphine V. Wegner,[1,2]* Oya I. Sentürk[1,2] & Joachim P. Spatz[1,2]

[1] Department of New Materials and Biosystems, Institution Max Planck Institute for Intelligent Systems, Heisenbergstr. 3, 70569 Stuttgart, Germany.

[2] Department of Biophysical Chemistry, University of Heidelberg, Im Neuenheimer Feld 253, 69120 Heidelberg, Germany.

E-mail: wegner@is.mpg.de

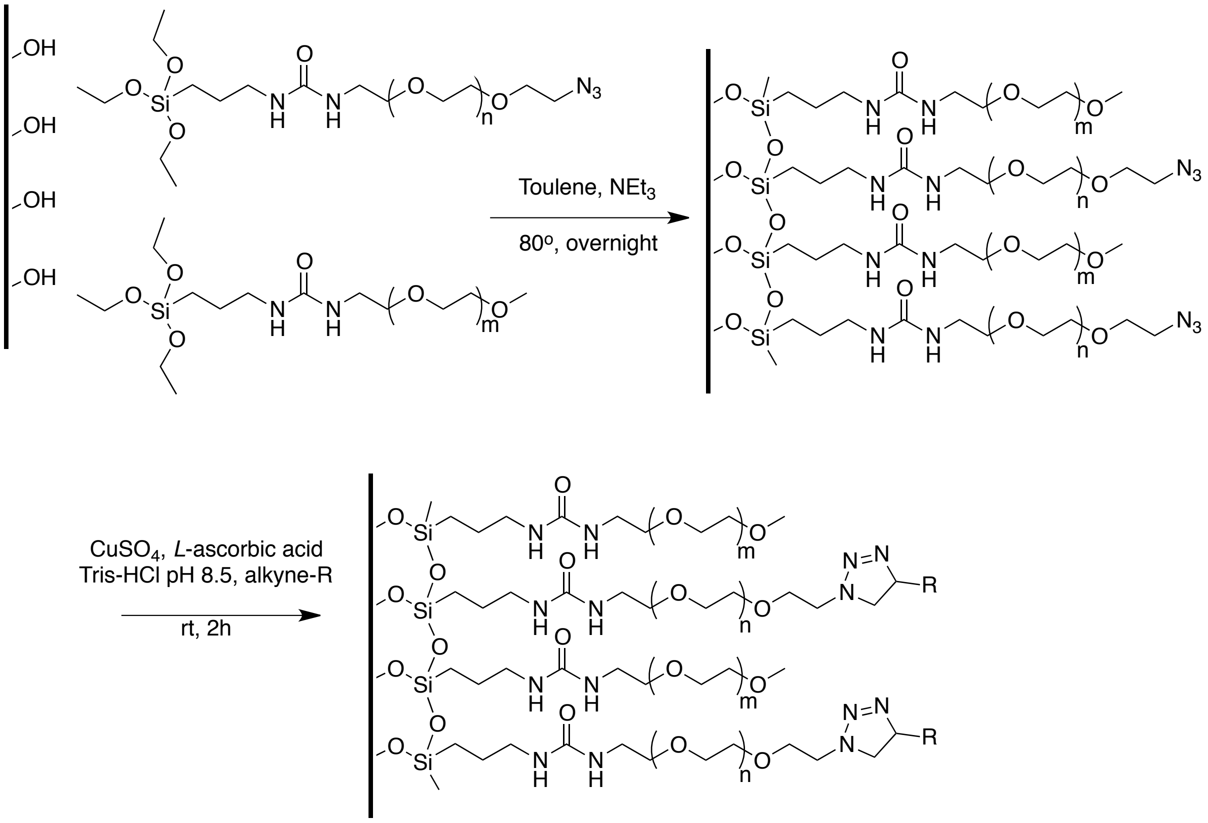

Figure S1. PEG-azide coating of SiO_2 surfaces and click-reaction at the surface.

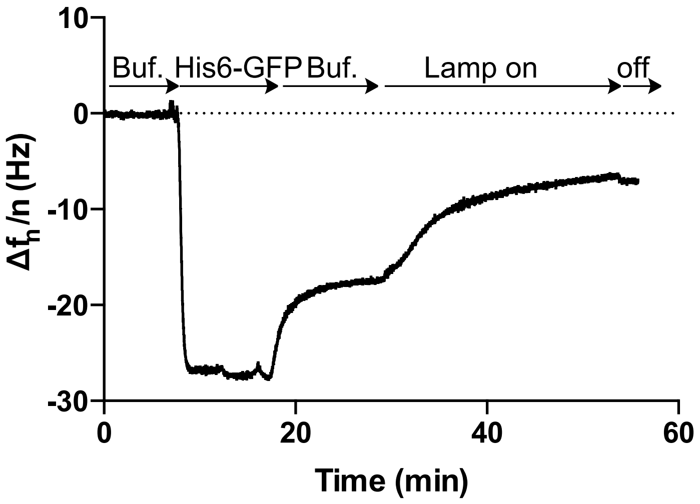

Figure S2. His6-GFP binds on SiO_2 QCM crystals coated with 100 % PEG-azide modified with NTA-NO_2. When the lamp is turned on the His6-GFP is liberated from the surface.

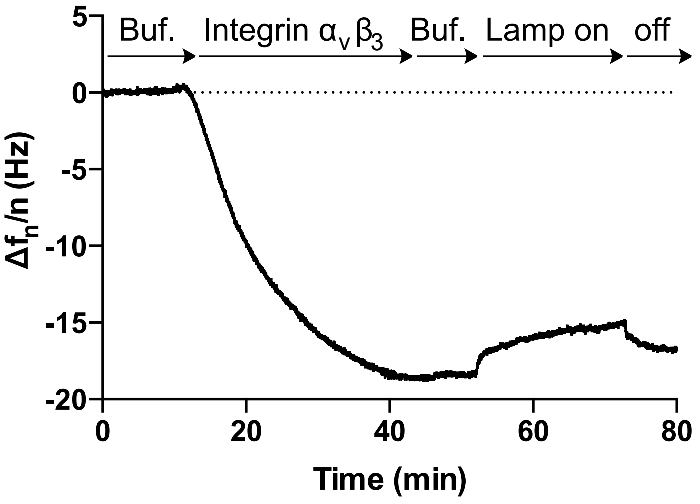

Figure S3. QCM measurements showing integrin $\alpha_v\beta_3$ binding to cRGD-modified 1 % PEG-azide surfaces. Unlike on cRGD-NO_2 modified surfaces, the bound integrin $\alpha_v\beta_3$ is not washed off from the cRGD-modified surface upon irradiated (λ = 365 nm).
